# Structural Characteristic and In-Vitro Anticancer Activities of Dandelion Leaf Polysaccharides from Pressurized Hot Water Extraction

**DOI:** 10.3390/nu15010080

**Published:** 2022-12-24

**Authors:** Pei Chen, Suyun Ding, Zhiqian Yan, Huiping Liu, Jianqiu Tu, Yi Chen, Xiaowei Zhang

**Affiliations:** State Key Laboratory of Food Nutrition and Safety, Key Laboratory of Food Nutrition and Safety, Ministry of Education of China, College of Food Engineering and Biotechnology, Tianjin University of Science & Technology, Tianjin 300457, China

**Keywords:** dandelion leaf, polysaccharides, structure, NMR, cell apoptosis

## Abstract

Dandelion (*Taraxacum mongolicum Hand.-Mazz.*) is a medicinal and edible plant. Dandelion has great development value for its health promoting benefits; additionally, Dandelion grows almost anywhere in the world. In this study, we report the structural characteristics and anti-cancer activity of novel dandelion leaf polysaccharides extracted by pressurized hot water extraction at 120 °C (DLP120) with Mw relative to dextran of 1.64 × 10^6^ Da. Structural analysis indicated that DLP120 is a complex polysaccharide composed of pectin and arabinogalactan. It was mainly composed of arabinose (32.35 mol%) and galactose (44.91 mol%). The main glycosidic linkages of DLP120 were 4-β-D-Gal*p*, 4-α-D-Gal*p*A, T-β-D-Gal*p*, 5-α-L-Ara*f*, 3,5-α-L-Ara*f*, and T-α-L-Ara*f*. In vitro, DLP120 inhibited HepG2 cell proliferation in a dose-dependent manner by inducing cell apoptosis. Cell cycle detection results revealed that DLP120 mainly arrests the cell cycle in S phase. Cells treated with DLP120 displayed obvious apoptotic morphology, including cell volume shrinks and cytoskeleton breaks down. In short, DLP120 has potential as an anti-cancer agent.

## 1. Introduction

Hepatocellular carcinoma (HCC) is one of the most common malignancies and causes of cancer death worldwide [1]. Almost one-third of patients use complementary medicines or herbal products to relieve symptoms and prevent relapse [2,3]. The effectiveness of therapeutic or chemopreventive drugs derived from plants have been reported [4,5]. Dandelion, a member of the Asteraceae family, is a perennial medicinal and edible herb. Its flowers, leaves, and roots have been used in traditional Chinese medicine [5,6]. Studies have demonstrated further that dandelion extracts from whole or parts of dandelion can preferably promote healthy properties [7,8,9,10]. The active ingredients extracted from different parts of dandelion mainly belong to the sesquiterpenoid, triterpenoid, phytosterol, flavonoids, and phenolic acid families, among others [11]. Dandelion extract has been widely used for its diuretic, blood sugar lowering, antimicrobial, anti-inflammatory, and appetite stimulating properties [3,12].

Studies have shown that plant polysaccharides have many pharmacological activities such as lowering blood sugar, reducing inflammation, and inhibiting tumor growth [13,14]. Plant polysaccharides are one of the most abundant biological macromolecules with a wide range of health-promoting properties in nature. They have a considerable application value due to their strong bioactivity, low cytotoxicity, and high safety profile. Polysaccharides have also received considerable attention in the research of dandelion extracts. So far, polysaccharides extracted from dandelion have been found to have various medicinal properties, exhibiting antioxidant, hypoglycemic, and modulating the gut microbiota [15,16].

The biological activity of polysaccharides can be greatly impacted by the extraction conditions [17,18]. Pressurized hot water extraction (PHWE) is a relatively new extraction technology for obtaining bioactive compounds from natural sources [19]. On the one hand, the surface tension and viscosity of the solvent decrease with the increase of temperature, and the diffusion coefficient increases. On the other hand, high temperature reduces intermolecular interaction. Therefore, PHWE may lead to a more complete and faster extraction process. In recent years, PHWE has been widely used in the extraction of bioactive polysaccharides. Polysaccharides extracted from waste coffee grounds using PHWE have good antioxidant and hypoglycemic activity [20]. Sakdasri et al. revealed that PHWE can be used as an efficient method for extracting active substances from dried mushrooms [21]. The PHWE method was also found to be an efficient method for extracting polysaccharides from sweet tea [22]. The extraction temperature of dandelion is almost always below 100 °C. However, research on the structure–effect relationship of polysaccharides above 100 °C is still limited. The main purpose of this study was to research the detailed structural characteristics and inhibitory effect on the proliferation of HepG2 in vitro of dandelion polysaccharides extracted at 120 °C to improve the comprehensive utilization of dandelion.

## 2. Materials and Methods

### 2.1. Materials

Taraxacum mongolicum leaves were purchased from Daxinganling Huitong Natural Products Development Co., Ltd., (Heilongjiang, China).HepG2 cells were purchased from the cell bank of Beijing Union Medical College Hospital. Human liver HL-7702 cells were purchased from Shanghai Cell Bank of Chinese Academy of Sciences. Unless otherwise specified, all chemicals and reagents were analytical grades. All test kits and T-series dextrans were purchased from Beijing Solable Technology Co., Ltd., (Beijing, China).

### 2.2. Extraction and Purification

The dandelion leaves dried to constant weight in an oven (50 °C) were crushed through an 80 micron mesh sieve to obtain dandelion powder. First, deionized water (2000 mL) was added to 100 g of dandelion powder and extracted at 120 °C, heating with autoclave for 2 h. It was concentrated by vacuum rotary evaporation to one-fifth of the original volume followed by filtration. Polysaccharides were precipitated with 60% ethanol concentration. Samples were incubated overnight at 4 °C, followed by centrifugation to collect polysaccharides. Extracts were deproteinized using the Sevag method [23]. The resulting solution was loaded into a 10 kDa dialysis bag and dialyzed to remove impurities and small molecules. The crude polysaccharide obtained by freeze-drying was dissolved and loaded onto a Sephadex G-200 gel column (16 mm × 40 cm). We refer to the resulting polysaccharide component as DLP120.

### 2.3. Chemical Composition Analysis

The phenol-sulfuric acid method [24], m-hydroxydiphenyl colorimetric method [25], and Bradford’s method [26] were used to determine the total sugar, uronic acid, and protein content with D-glucose, galacturonic acid, and bovine serum albumin serving as standards, respectively. UV spectroscopy (UV-2500PC, Shimadzu, Kyoto, Japan) was used to detect the protein and nucleic acid content of DLP120.

### 2.4. Molecular Weight Analysis

High performance liquid chromatography (HPLC) instrument (Agilent-1200, Agilent, Santa Clara, CA, USA) equipped with a TSK-gelG 4000 PWxl column (7.8 mm × 300 mm, column temperature 30 °C) and a refractive index detector (detector temperature 35 °C) was used to measure the average molecular weight of DLP120. The sample (1 mg/mL) injection volume was 20 μL using ultrapure water as the mobile phase. The molecular weight of DLP120 was estimated by a calibration curve obtained from T-series dextran standards (T10, T40, T70, T110, T500, and T2000 kDa).

### 2.5. Monosaccharide Composition Analysis

Ion chromatography (IC) instrument (Thermo Dionex ICS2500, Thermo, Waltham, MA, USA) was used to detect the monosaccharide composition of polysaccharides of DLP120 through a high-efficiency anion exchange chromatography equipped with a Dionex pulsed amperometric detector with Au electrodes and a Dionex Carbopac PA10 column (150 mm × 3 mm). The sample (1 mg) was hydrolyzed in 2 M trifluoroacetic acid (TFA) in an oil bath at 110 °C for 3 h.

TFA was removed by blowing N_2_ with methanol. The composition and content of monosaccharides were determined based on the retention time and peak area of monosaccharide standards (rhamnose, arabinose, galactose, glucose, xylose, mannose, galacturonic acid, and glucuronic acid).

### 2.6. Fourier Transform Infrared Spectroscopy (FT-IR) Analysis

The absorption spectrum of DLP120 was scanned by infrared spectrometer (Bruker VECTOR-22, Karlsruhe, Germany) with the detector DTGS. Briefly, 1 mg sample and 150 mg KBr were ground into powder and pressed into tablets for analysis. The infrared spectra of DLP120 were obtained by scanning 16 times in the range of 4000–400 cm^−1^ with a resolution of 4 cm^−1^.

### 2.7. Reduction of Uronic Acids

The polysaccharide sample (5 mg) was dissolved in distilled water (2 mL). The solution was mixed with 1-(3-Dimethylaminopropyl)-3-ethylcarbodiimide hydrochloride while adding 0.1 M HCl to adjust the pH to 4.75 stirring continuously for 1 h. 5 mL of sodium borohydride solution (160 mg/mL) was added dropwise over a period of 1 h. The pH of the solution was adjusted to 7.0 by slowly adding 2 M HCl and stirring for 0.5 h. After the pH of the solution was brought back to 4.0, it was dialyzed with distilled water, and vacuum freeze-dried. The polysaccharide was redissolved in 10% acetic acid in methanol solution and blown dry with N_2_ to remove boric acid, which was repeated four times. Finally, methanol solution (0.5 mL) was added and dried by N_2_ (three times) to obtain the reduced polysaccharide.

### 2.8. Methylation Analysis

The reduced sample was fully dissolved in dimethyl sulfoxide (DMSO), and then dried NaOH powder was added for 30 min of ultrasonic reaction to ensure a sufficient dissolution. Methyl iodide was added and reacted for 1 h at 18–20 °C under sonication and dark conditions followed by adding distilled water to stop the methylation reaction. The methylation reaction solution was extracted with dichloromethane to obtain a methylated product. Hydrolyzed samples were obtained by adding 2 M trifluoroacetic acid (TFA) to the sample, heating at 110 °C for 3 h, followed by drying with N_2_, and redissolving the sample in distilled water. The NaBD_4_ was added to reduce the hydrolysate, followed by acetylating with acetic anhydride for 2 h. The resulting partially methylated alditol acetates (PMAA) were analyzed by GC-MS (BRUKER 456GC TQ; column: BR 1701). Three parallel groups were set up.

### 2.9. Nuclear Magnetic Resonance (NMR) Spectroscopy

DLP120 (70 mg) was dissolved in 0.5 mL D_2_O at 25 °C, assisted by ultrasound. The ^1^H, ^13^C, ^1^H/^1^H COSY, ^1^H/^1^H TOCSY, ^1^H/^13^C HSQC ^1^H/^13^C HMBC NMR spectra were recorded on a Bruker AVANCE Ⅲ HD 700 MHz spectrometer with a BBO (Broadband Observe) cryoprobe. Detailed NMR experiment parameters are shown in Table S1.

### 2.10. Anticancer Activity In Vitro

#### 2.10.1. Cell Culture

HepG2 human liver cancer cells were cultured in DMEM complete medium (10% (*v*/*v*) fetal bovine serum and 1% penicillin/streptomycin) and kept at 5% CO_2_ completely humidified at 37 °C. Cells were seeded in 96-well plates or 6-well plates for biological analysis. The DLP120 group was composed of lyophilized DLP120 as solute and cell culture medium as solvent configured into different concentrations of samples. The control group was the cell culture medium without DLP120. The cells were treated with different concentrations of DLP120 and placed in a cell incubator for 24 h.

#### 2.10.2. Cell Viability and Proliferation

The effect of DLP120 on HepG2 and HL-7702 cell viability was determined by MTT (3-(4,5-dimethylthiazolyl-2)-2, 5-diphenyltetrazoliumbromide) reagent. Cells in the 96-well plate were treated with different concentrations of DLP120 (0, 200, 400, 600, 800, 1000 μg/mL) for 24 h, followed by addition of MTT. The blank group was cell culture medium without cells. The absorbance value was measured at 570 nm with a microplate reader (Bio-Rad, Hercules, CA, USA). The inhibitory rate following the addition of DLP120 was calculated using the following formula:

(1) $$\mathrm{cell}\;\mathrm{viability}\;\left(\%\right)=\frac{{\mathrm{OD}}_{1}-{\mathrm{OD}}_{0}}{{\mathrm{OD}}_{2}-{\mathrm{OD}}_{0}}\times 100$$

where OD_0_ is the OD value of the blank group, OD_1_ is the OD value of the DLP120 group, and OD_2_ is the OD value of the control group.

#### 2.10.3. Observation of Cell Morphology by Scanning Electron Microscope (SEM)

HepG2 cells were inoculated into 6-well plates by climbing slides. The cells were cultured with different concentrations of DLP120 for 24 h. Cell images were obtained using an inverted light microscope (Nikon, Tokyo, Japan) to observe changes in cell morphology. After the end, cells were fixed with 2.5% glutaraldehyde for 4 h, then dehydrated with gradient ethanol, and cell morphology was observed using SEM (Hitachi, Tokyo, Japan).

#### 2.10.4. Apoptosis Assay

HepG2 cells were treated with DLP120 for 24 h in 6-well plates. Then, the cells were washed and fixed with 4% paraformaldehyde. Cells were stained with Hoechst 33258 (25 μL) and incubated for 20 min at room temperature in the dark. Finally, the stained cells were observed under an inverted fluorescence microscope (Nikon ECLIPSE 90i, Tokyo, Japan).

#### 2.10.5. Cell Cycle Assay

The cell cycle was conducted by flow cytometry (Becton Dicknson, Franklin Lakes, NJ, USA). Cells treated with DLP120 for 24 h were collected and fixed overnight with ice-cold 70% ethanol. The assay was conduction following the manufacturer’s protocol, adding propidium iodide (PI) staining solution to detect cell death by CellQuest Pro6.

#### 2.10.6. Annexin V-FITC Assay

HepG2 cells were collected after 24 h treatment with DLP120. The cells were resuspended with binding buffer and normalized to 5 × 10^6^ cells/mL. Annexin V/FITC staining solution was added for 5 min followed by addition of PI staining solution. Samples were assayed using flow cytometry as soon as possible.

### 2.11. Statistical Analysis

All experimental data were statistically evaluated by using Student’s *t*-test and one-way ANOVA. Each experiment was repeated at least three times, and the results were expressed as mean ± standard deviation.

## 3. Results

### 3.1. Chemical Compositions

**Figure 1:**
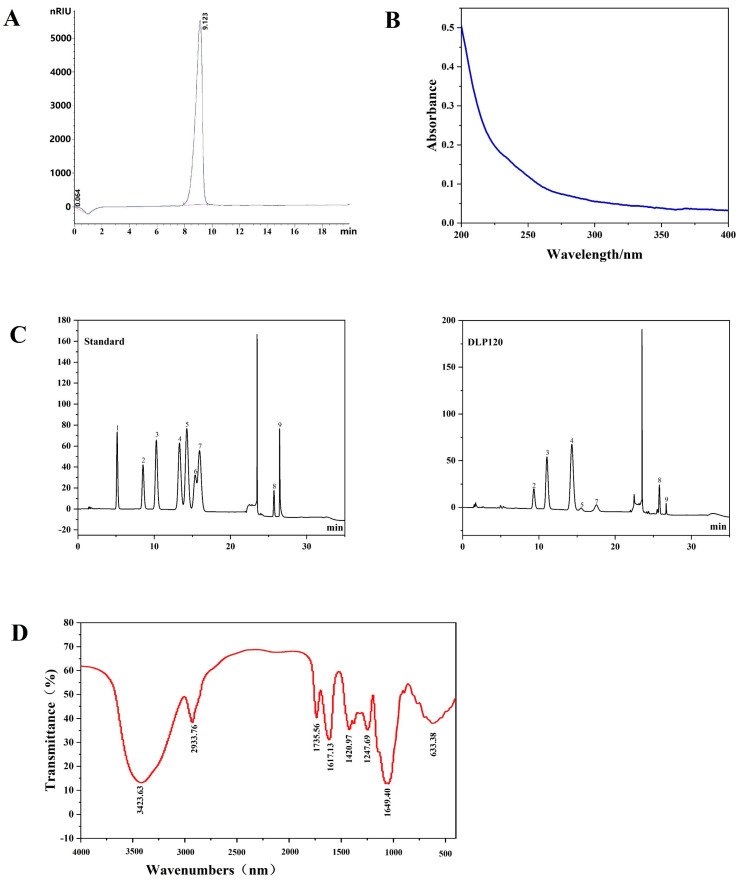
(**A**) The HPLC spectrum of DLP120. (**B**) UV spectrum of DLP120. (**C**) IC of the standard monosaccharides and the monosaccharides derived from DLP120: (1) fucose; (2) rhamnose; (3) arabinose; (4) galactose; (5) glucose; (6) xylose; (7) mannose; (8) galacturonic acid; (9) glucuronic acid. (**D**) FT-IR spectrum (in KBr) of DLP120.

The results showed that the contents of total sugar, protein, and uronic acid percentages of 92.1%, 1.52%, and 14.21%, respectively. DLP120 extraction at 120 °C resulted in a higher yield (7.24%) than the extraction temperature of 80 °C (3.57%) following the previous extraction method [27]. It revealed that temperature had a significant positive effect on the yield of water-soluble polysaccharides from dandelion consistent with previous reports [28,29]. HPLC of crude polysaccharide is in Figure S1. The molecular weight (Mw) of DLP120 was measured by HPLC using dextran as a standard. As shown in Figure 1A, DLP120 showed a narrowly dispersed symmetrical peak on HPLC. The Mw relative to dextran of DLP120 measured by HPLC was 1.64 × 10^6^ Da (based on the calibration curve y = −0.3434x + 9.3478, R^2^ = 0.9914), which differs significantly from previous reports about small-molecule polysaccharides [30,31]. As shown in Figure 1B, UV full-wavelength scanning confirmed that the DLP120 contained seldom nucleic acids or proteins.

### 3.2. Monosaccharide Composition

The monosaccharide composition and the molar ratio of DLP120 were identified by IC analysis. The results showed that DLP120 mainly consisted of rhamnose, arabinose, galactose, mannose, and galacturonic acid in a molar ratio of 1.95:6.37:8.84:1:1.04 (Figure 1C). In addition, DLP120 also contained a small amount of glucose (0.29) and glucuronic acid (0.19). Monosaccharide standard curve of DLP120 is in Table S2.

### 3.3. FT-IR Analysis

FT-IR analysis was also conducted on DLP120. The sample displayed a wide and strong absorption band at 3416 cm^−1^ and indicated the presence of O-H tensile vibration (Figure 1D). The absorption bands around 2931 cm^−1^ were derived from the presence of C-H tensile vibration [6]. The strong band from 1617 cm^−1^ could be assigned to the absorbance of C=O bond tensile vibration. The absorption bands in the range of 1400–1200 cm^−1^ might be attributed to the bending of C-H bond [32]. The characteristic bands near 1046 cm^−1^ and 500–800 cm^−1^ have been associated with the presence of pyranose rings [33,34]. These absorption bands were typical infrared signals of polysaccharides. We also observed a stretching vibration of DLP120 at 1735 cm^−1^, suggesting the presence of a uronic acid structure. This analysis further confirmed that DLP120 is an acidic polysaccharide, consistent with the IC results.

### 3.4. Methylation Analysis

**Figure 2:**
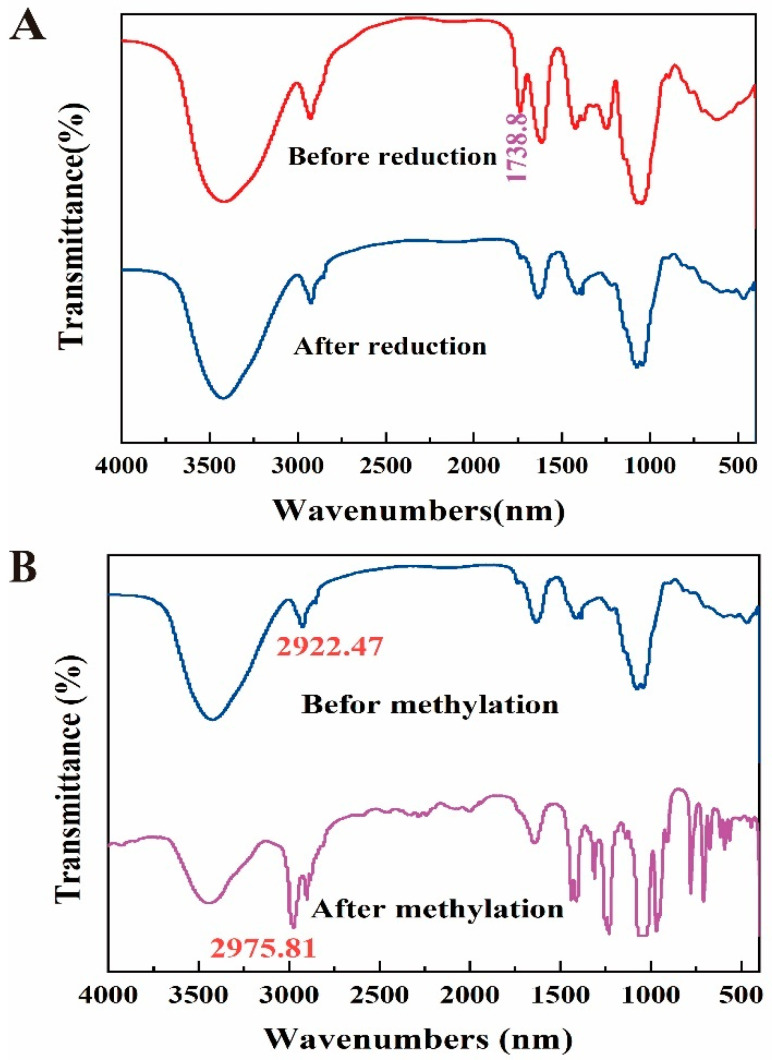
(**A**) FT-IR spectra of DLP120 before and after reduction. (**B**) FT-IR spectra of DLP120 before and after methylation.

To address the concern that the link between the uronic acid and neutral sugar moieties may have been lost, we first reduced the carboxyl group of DLP120 before methylation analysis. As measured in the FT-IR, the carboxyl group in DLP120 uronic acid was successfully reduced (Figure 2A) and methylated completely (Figure 2B). The GC-MS chromatogram analysis of DLP120 showed a complex structure with 10 linkage patterns. Sugar residues and PMAA of DLP120 are in Figures S2 and S3, respectively. The methylation analysis was shown in Table 1 (listed in order of monosaccharide composition). The most abundant peak was derived from the residues of 4-Gal*p* (40.29%). There were two main derivatives, T-Gal*p* (11.14%) and 5-Ara*f* (10.35%). Other sugar residues including T-Ara*f* (7.46%), 3,5-Ara*f* (7.51%), 2,4-Rha*p* (6.88%), T-Rha*p* (0.86%), 6-Gal*p* (4.95%), 4-Man*p* (6.35%), and T-Glc*p* (4.19%) were observed as well. It should be noted that the use of NaBH_4_ instead of NaBD_4_ for the reduction before methylation made it impossible to distinguish Gal*p*A from Gal*p*.

Noticeably, in the composition which was inferred from the methylation analysis differed with the monosaccharide compositional analysis. This difference may be caused by the hydrolysis process [35,36]. The monosaccharide composition is directly hydrolyzed, while the methylation experiment is hydrolyzed after the methylation reaction. In addition, the ratio of branching groups to end groups in the methylation is not equal. On the one hand, it may be due to the incomplete hydrolysis process caused by the end groups being hydrolyzed first and the branched chain residues being hydrolyzed afterwards during hydrolysis [35,37]. On the other hand, it may be on account of the presence of a small number of oligosaccharides in the sample, which participate in the methylation process leading to high end group.

### 3.5. NMR Analysis

**Figure 3:**
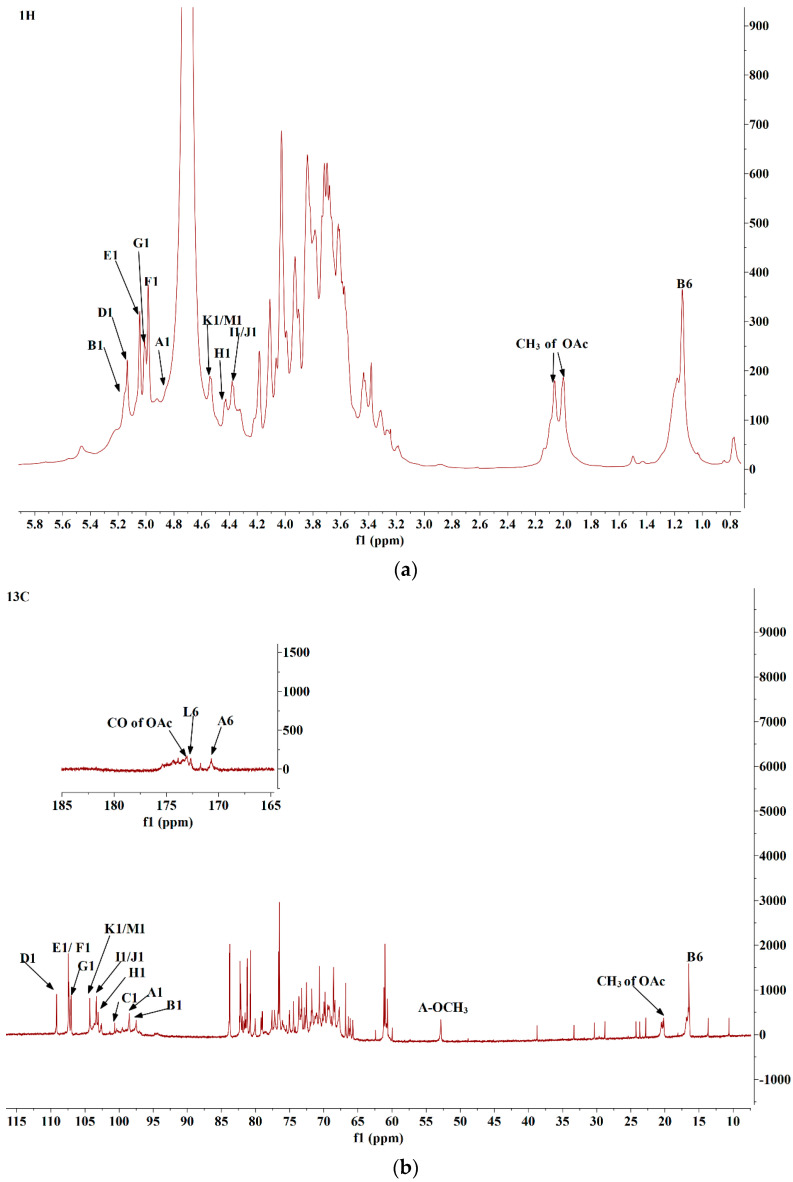

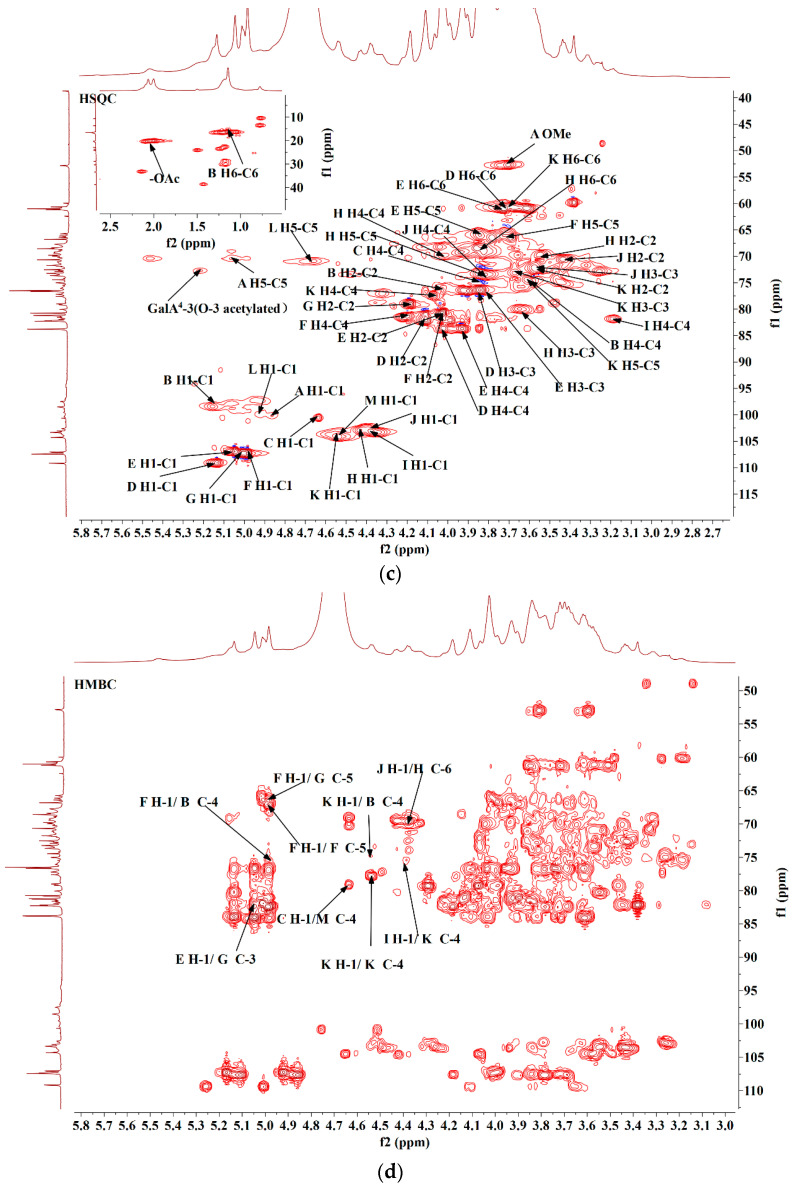

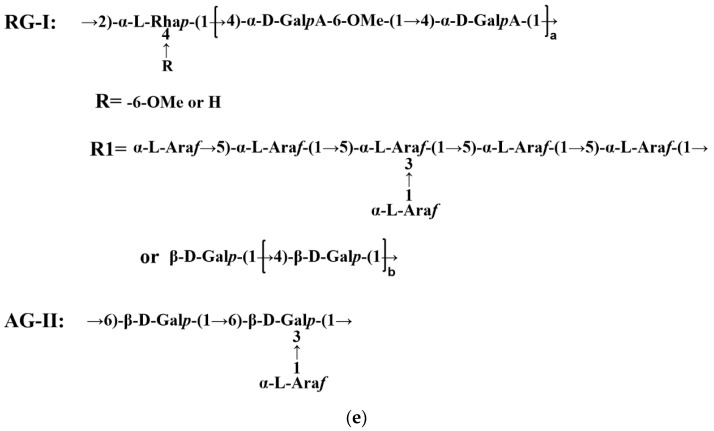
^1^H (**a**), ^13^C (**b**), HSQC (**c**), HMBC (**d**) spectra of DLP120 and structural model (**e**) of DLP120. Note: In (**e**), the main chain residue of RG-I 4-α-Gal*p*A has a small amount of O-3 acetylation substitution; in addition, the sample may also exist residue fragment of α-L-Rha*p*-(1→4)-β-Glc*p*A-(1→).

The NMR spectra (including ^1^H, ^13^C, COSY, TOCSY, HSQC, and HMBC) were obtained to determine the glycoside linkages of DLP120 in detail. From 1D spectra (Figure 3a,b), the most intensive and sharp peak (3.70/52.29 ppm) was attributed to the methoxyl group [38]. The signals were assigned to methyl and acetyl groups at 1.14 and 1.97 ppm (Figure 3a), respectively. The methyl group was derived from rhamnose (C-6). This deduction also was verified by the cross peaks in HMQC with the chemical shift of 1.14/16.92 ppm. The cross peaks in the HSQC (1.97/20.22 ppm) (Figure 3c) and HMBC (1.97/173.29 ppm) (Figure 3d) spectrum confirmed the presence of the -OAc groups [39]. The ^13^C signals at 170–175 ppm (Figure 3b) were attributed to the C-6 of uronic acid by comparison to previous analyses [38].

The NMR spectrum (Figure 3a) showed thirteen significant peaks in the anomeric region. Particularly, a new peak (C) could be clearly observed for the HMQC (Figure 3c) in addition to the twelve overlapping peaks. All peaks in the anomeric region were assigned as A-M at 4.86, 5.17, 4.63, 5.13, 5.04, 4.99, 5.01, 4.43, 4.38, 4.37, 4.54, 4.92, and 4.56 ppm. The corresponding ^13^C assignments were achieved according to HSQC. The complete ^1^H assignments were acquired through COSY and TOCSY spectra (Figure S4a,b). Assignments of ^1^H and ^13^C based on methylation results and comparison to literature data are shown in Table 2.

The signals at δ 4.86/100.18 were confirmed as α-configuration of residue A (Figure 3a,b). The H-2-H-5 signals of residue A were assigned through the cross peaks of COSY spectra (Figure S4a). Their chemical shifts were attributed to 3.64, 3.90, 4.31, and 5.06 ppm. The chemical shift of C-1-C-5 of residue A can be attributed by HSQC spectrum (Figure 3c), which were 100.18, 68.07, 69.02, 77.22 and 70.35 ppm. The HSQC spectrogram (Figure 3c) showed that the methyl proton signal and carbon signal of methyl ester are 3.70 ppm and 52.29 ppm, respectively. The chemical shift of C-1 and C-4 shifted to the lower field, indicating that the residue was replaced at the positions of C-1 and C-4. Combining the methylation results and literature reports [40], it was inferred that the residue A was 4-α-Gal*p*A-6-OMe.

The anomeric chemical shift of ^1^H at 5.17 ppm and ^13^C 98.43 ppm demonstrated that residue B should be assigned to rhamnose residue. The chemical shifts of H-1 (5.17 ppm) and H-6 (1.14 ppm) and of residue B were resolved by ^1^H NMR. According to the COSY spectrum, the H-2-H-5 shifts of residue B were attributed to 4.04, 3.84, 3.60 and 3.74 ppm. By HSQC correlation spectrum (Figure 3c), the chemical shifts of each carbon were attributed to be 98.43, 76.11, 68.65, 75.11, 67.28 and 16.92 ppm. It was inferred that residue B was 2,4-α-L-Rha*p* based on a comparison to existing literature [39].

The anomeric proton at 4.63 ppm was correlated to the anomeric carbon signal at 100.61 ppm, which designated residue C. It was worth noting that this signal clearly appeared in COSY and HSQC (Figure S4a and Figure 3c), but was not reflected in the ^1^H spectrum. Presumably, the signal was masked by the solvent peak (D_2_O). Based on these observations, it was inferred that residue C was T-α-L-Rha*p* [41].

Residue D was attributed as T-α-L-Ara*f* according to their anomeric chemical shifts at 5.13 ppm and 109.18 ppm (Figure 3a,b). The H chemical shift of sugar residue D was determined by the cross peak of COSY and TOCSY spectrum (Figure S4a,b). The chemical shift of carbon could be assigned by HSQC spectrum (Figure 3c). The C-1 shifted to a lower field, indicating that the residue was replaced at sugar ring position C-1 [41,42]

The 5.04 and 107.09 ppm signals indicate that residue E was the α configuration. From the COSY and TOCSY spectrum (Figure S4a,b), the complete ^1^H assignments were obtained. Corresponding carbon shifts could be assigned by HSQC correlation spectra (Figure 3c). Similar to residue D, it was inferred that residue E was also T-α-L-Ara*f* [41].

Residue F (5-α-L-Ara*f*) and G (3,5-α-L-Ara*f*) showed anomeric chemical shifts at δ 4.99/5.01 (Figure 3a) and δ 107.43/107.32 (Figure 3b) according to ^1^H and ^13^C. All assignments of ^1^H by COSY and TOCSY spectra and ^13^C by the HSQC spectrum were also shown in Table 2. Integrating with methylation and literature data [41], residue F was inferred to be 5-α-L-Ara*f* and residue G was inferred to be 3,5-α-L-Ara*f*.

The 4.43 ppm (H-1) and 103.14 ppm (C-1) signals indicated that H was in the β configuration. The H-2-H-5 chemical shifts of residue H were attributed to 3.54, 3.64, 4.01 and 3.81 ppm by the cross-peak of COSY spectrum (Figure S4a). The chemical shift of carbon could be assigned by the HSQC correlation spectrum (Figure 3c). Residue H was inferred to be 3,6-β-D-Gal*p* [41].

The chemical shift at 4.38 ppm and 4.37 ppm (Figure 3a) and the ^13^C chemical shift at 103.21 ppm and 103.25 ppm (Figure 3b) were tentatively derived from T-β-D-Gal*p* (residue I) and 6-β-D-Gal*p* (residue J), respectively. The complete ^1^H assignments were acquired through the COSY and TOCSY spectrum. The chemical shifts of all carbons were assigned through the HSQC correlation spectrum (Figure 3c), which are shown in Table 2 [41].

Methylation analysis indicated that there is a substantial presence of 4-D-Gal*p/*Gal*p*A in DLP120, with the highest mole ratio of 40.29%. The integral at 4.54 ppm in the ^1^H spectrum was significantly higher than other signals. Combining the analysis of monosaccharide composition, methylation results, and the previous studies [38,39], the anomeric chemical shift of δ 4.54/104.33 in the anomeric region was classified as 4-β-D-Gal*p* (K). The attribution of ^1^H was obtained by COSY. Moreover, three peaks at 3.57, 3.66, 4.54 ppm were also obtained in the TOCSY spectrum (Figure S4b). ^13^C chemical shifts of 4-β-D-Gal*p* were achieved by HMQC.

The chemical shift at 4.92 ppm (H-1) and 99.71 ppm (C-1) was named residue L. We concluded that the sugar residue L was 4-α-Gal*p*A by comparison of chemical shifts to data in the literature [16,39]. The anomeric carbon signal at 73.23 ppm was correlated to the anomeric proton at 5.22 ppm, suggesting that the acetyl group was linked to 4-α-Gal*p*A at the O-3 position [43,44].

4-β-Glc*p*A (residue M) exhibited anomeric chemical shifts at 4.56/104.00 ppm. The complete assignments were obtained from COSY and HSQC, which were consistent with previous reports [41].

Through observing inter- and intra-residual connectivity in the HMBC spectrum (Figure 3d), the sequence of linkages between sugar residues was deduced. The H1 (4.99 ppm) signal peak of residue F correlated with the three sugar residues at the C-4 (75.11 ppm) of residue B (F H-1/ B C-4), C-5 (66.04 ppm) of residue G (F H-1/ G C-5), and C-5 (66.20 ppm) of residue F (F H-1/ F C-5). The H1 (5.04 ppm) signal peak of residue E correlated with the C-3 (82.22 ppm) of residue G (E H-1/G C-3). The H1 (4.37 ppm) signal peak of residue J correlated with the C-6 (69.26 ppm) of residue H (J H-1/ H C-6). The H1 (4.54 ppm) signal peak of residue K was related to the residues at the C-4 (75.11 ppm) of residue B (K H-1/B C-4). Correlations were between the H1 (4.63 ppm) of residue C and C-4 (79.02 ppm) of residues M (C H-1/ M C-4). The H1 (4.38 ppm) signal peak of residue I was related to the sugar residues at the C-4 (77.77 ppm) of residue K (I H-1/K C-4). In particularly, intra-residue cross peaks were also observed between H-1 (4.54 ppm) of the residue K and its own C-4 (104.33 ppm).

Combining the monosaccharide composition, methylation results, and NMR (1D and 2D) information analysis of the polysaccharide samples, the possible structural models of the DLP120 were inferred as shown in Figure 3e [45,46].

### 3.6. Anticancer Activity In Vitro

#### 3.6.1. Effect of DLP120 on HepG2 Cell Proliferation

**Figure 4:**
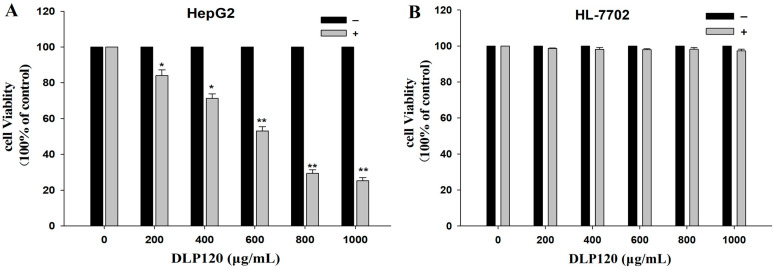
MTT results of DLP120 on HepG2 cells (**A**) and HL-7702 cells (**B**) at 24 h. * *p* < 0.05 ** *p* < 0.01 vs. control.

We used the MTT reagent to measure cell viability. As shown in Figure 4A, DLP120 exhibited an inhibitory effect on HepG2 in a dose-dependent manner. Compared with HepG2 cells, the viability of HL-7702 cells was not significantly changed (Figure 4B). Thus, DLP120 has no toxic effect on normal liver cells. The cell viability decreased from 84.0% to 71.3%, while the concentration of DLP120 increased from 200 μg/mL to 400 μg/mL. Moreover, at a dose of 800 μg/mL, DLP120 showed a tumor suppressive effect on HepG2 cells, with a cell viability of 29.4%. Feng et al. demonstrated that treatment of Eca109 cells with 2 mg/mL dose of Ganoderma lucidum polysaccharides accelerated the apoptosis of Eca109 cells through the mitochondrial pathway with a proliferation inhibition rate of 74.63% [47]. Gamargo et al. revealed that only 10 mg/mL and 5 mg/mL of Ganoderma lucidum polysaccharides significantly reduced tongue squamous cell metabolism, resulting in non-invasive and less proliferative behavior of tumor cells [48]. Together, these results indicated that DLP120 has a certain potential to inhibit the proliferation of HepG2 cells.

#### 3.6.2. Effect of DLP120 on HepG2 Cell Morphology

**Figure 5:**
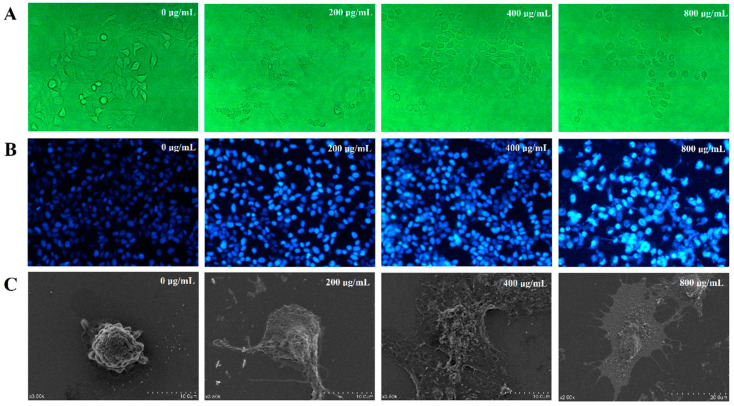
(**A**) Cell morphology in HepG2 treated and untreated cells was observed by inverted microscope. (**B**) Apoptosis was detected by Honest 33258 staining. (**C**) Cell morphology was observed by SEM.

We next analyzed the cell morphology of treated and untreated HepG2 cells by microscopy. Untreated HepG2 cells displayed intact cell membranes as expected (Figure 5A,C). In addition, the surface microvilli were not only rich but also complete on the surface. The cells were arranged regularly and mosaic-connected, as expected for normal HepG2 cells. Cell morphology was altered dramatically for cells treated with different concentrations of DLP120. Among the observed effects were the shrinking of cell volume, chromatin condensation, the disappearance of the tightly connected structure between cells, and the fragmentation of cytoskeletal disintegration. All the above effects indicate that treatment with DLP120 promoted apoptosis in HepG2 cells.

The results of Hoechst33258 staining further confirmed that HepG2 cells showed typical apoptotic morphological characteristics after DLP120 treatment. As can be seen from Figure 5B, the HepG2 nuclei of the control group had regular morphology and were uniformly stained blue, while those treated with DLP120 showed nuclear pyknosis, chromatin condensation, nuclear debris, and apoptotic bodies. These phenomena became more and more pronounced with the increase of DLP120 concentration. The above results indicated that DLP120 induced apoptosis in HepG2 cells.

#### 3.6.3. Effect of DLP120 on HepG2 Cell Cycle Distribution

**Figure 6:**
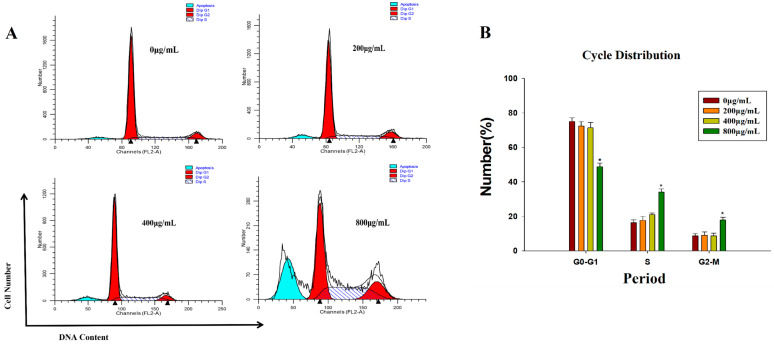
Cell cycle proportions of HepG2 cells treated with DLP120 for 24 h. (**A**) Bar graph summarizes the data on cell cycle distribution. (**B**) * *p* < 0.05 vs. control.

To determine whether DLP120 could cause cell cycle redistribution, the percentage of HepG2 cells in G1, S, and G2 phase was assessed by flow cytometry. As shown in Figure 6, the appearance of the apoptosis peak was evidenced by a clear accumulation of S phase cells. In addition, the proportion of cells in the G1 phase decreased while cells in the G2 phase increased. Therefore, we conclude that DLP120-induced apoptosis was accompanied by cell cycle arrest in the S phase of HepG2 cells, which could lead to inhibition of the proliferation of tumor cells. Similar studies have reported that components in some natural products displayed anticancer activity by preventing cell cycle distribution and inducing apoptosis [49].

#### 3.6.4. Effect of DLP120 on HepG2 Cell Apoptosis

**Figure 7:**
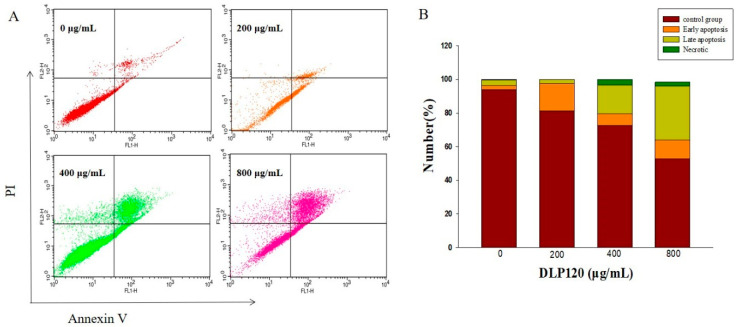
Distribution of apoptosis after cells treated with DLP120 (**A**). Bar graph summarizes the percentage of apoptosis (**B**).

Alterations from cell membrane surface integrity are a typical feature of early apoptosis. This change can be detected by flow cytometry using Annexin V and PI double staining. In our experiments, cells treated with varying concentrations of DLP120 showed different degrees of early and late apoptosis (Figure 7). Untreated cells primarily consisted of live cells in a normal state. The proportion of apoptotic cells increased in a dose-dependent manner. The total apoptosis rate increased dramatically from 18.80% to 47.13% as the concentration of DLP120 increased from 200 μg/mL to 800 μg/mL (*p* < 0.05). These data suggested that DLP120 could induce apoptosis of HepG2 cells. Combined MTT and cell cycle analysis revealed that DLP120 inhibited the proliferation of HepG2 cells by inducing apoptosis.

## 4. Discussion

In previous reports, several polysaccharide components of dandelion leaf extracted below 100 °C have been identified, including β-branched glucomannan [50], a neutral polysaccharide dominated by glucose and galactose [15], as well as acidic polysaccharides, which lack specific structural information on methylation and 2D NMR support [16]. In this study, we found that DLP120 consists of a complex of pectin (RG-Ⅰ) and arabinogalactan (AG-Ⅱ). Pectin-like substances have been rarely found in the literature on dandelion. Pectin itself has been shown to inhibit tumor growth and significantly reduce the rate of tumor metastasis [51]. Regarding the structure of DLP120, RG-Ⅰ appears methyl esterified and acetyl esterified in the carboxyl portion of C-6 and the hydroxyl portion of O-3 of GalA, respectively, which is important for the anti-cancer activities of pectic polysaccharides [45,52]. It has been shown that RG-Ⅰ, especially modified GalA, and the neutral sugar side chain AG-Ⅱ tend to interact with target substances to promote cell cycle arrest and induce apoptosis [53,54,55,56]. These functional structural regions (RG-Ⅰ) may inhibit the proliferation of cancer cells by reducing the expression of intercellular adhesion molecules [53], and AG-Ⅱ may interact with extracellular matrix proteins to induce apoptosis [57]. However, current studies on the specific recognition receptors of pectin polysaccharides and their activation mechanisms are insufficient, and systematic and comprehensive studies are still necessary [45,58].

In addition, pectic polysaccharides can selectively bind to galectin-3 (Gal-3), which is a potential target for cancer therapy and involved in cell proliferation, adhesion, and regulation of the cell cycle, to prevent and reduce cancer progression [59,60,61]. Galactans in pectin have been shown to directly bind Gal-3 [61]. DLP120 is a macromolecular pectin with abundant neutral sugar side chains of arabinogalactan. The monosaccharides are predominantly galactose and arabinose. On the one hand, larger molecular weight pectins with multiple galactose side chains may contain more Gal-3 binding sites compared to smaller molecular weights [59]. On the other hand, galactose side chains contained in pectins have a stronger binding capacity to Gal-3 compared to galactans alone [62]. Furthermore, RG-Ⅰ type pectins isolated from Panax ginseng flower buds containing abundant side chains had stronger binding activity to Gal-3 than HG structures [63]. Polysaccharides with different structural features have mechanisms involving various signaling pathways to exert anti-cancer effects. In our study, we found that DLP120 mainly arrest the cell cycle in the S phase to exert its anti-cancer activity. Due to its complex structure, it has been difficult to establish the specific structure of DLP120 and clarify its anti-cancer mechanism. In future work, new methods of elucidating the mechanism of DLP120 tumor inhibition are required.

## 5. Conclusions

In conclusion, an acidic water-soluble polysaccharide was extracted and purified from the leaves of dandelion at 120 °C with autoclave as DLP120. We described the structural characteristics and anti-cancer activity of DLP120. According to the analysis of HPLC, IC, methylation, as well as 1D and 2D NMR, DLP120 is a high molecular weight heteropolysaccharide with a complex structure mainly composed of RG-Ⅰ and AG-Ⅱ. In vitro experiments revealed that DLP120 inhibited the proliferation of HepG2 cells by arresting their cell cycle S phases.

## Figures and Tables

**Table 1:** GC-MS of alditol acetate derivatives from the methylated products of DLP120.

Types of Linkage	RT (min)	PMAA	Molar Ratio (%) ^a^
T-Rha*p*	18.182	1,5-O-Ac_2_-2,3,4-O-Me_3_-rhamnitol	0.86
2,4-Rha*p*	23.160	1,2,4,5-O-Ac_4_-3-O-Me-rhamnitol	6.88
Total			7.74
T-Ara*f*	17.565	1,4-O-Ac_2_-2,3,5-O-Me_3_-arabinitol	7.46
5-Ara*f*	21.107	1,4,5-O-Ac_3_-2,3-O-Me_2_-arabinitol	10.35
3,5-Ara*f*	26.664	1,3,4,5-O-Ac_4_-2-O-Me-arabinitol	7.51
Total			25.32
T-Gal*p*	21.455	1,5-O-Ac_2_-2,3,4,6-O-Me_4_-galactitol	11.14
4-Gal*p/*Gal*p*A	24.283	1,4,5-O-Ac_3_-2,3,6-O-Me_3_-galactitol	40.29
6-Gal*p*	25.212	1,5,6-O-Ac_3_-2,3,4-O-Me_3_-galactitol	4.95
Total			56.38
T-Glc*p*	22.017	1,5-O-Ac_2_-2,3,4,6-O-Me_4_-glucitol	4.19
Total			4.19
4-Man*p*	24.050	1,4,5-O-Ac3-2,3,6-O-Me_3_-mannitol	6.35
Total			6.35

RT: Retention time; PMAA: partially methylated alditol acetates; a: Relative molar ratio, calculated from the ratio of peak areas.

**Table 2:** The ^1^H NMR and ^13^C NMR chemical shifts for DLP120.

Sugar Residues		Chemical Shift (ppm)								
			1	2	3	4	5	6a	6b	CH_3_- of -OMe
A	4-α-Gal*p*A-6-OMe	H	4.86	3.64	3.9	4.31	5.06			3.7
		C	100.18	68.07	69.02	77.22	70.35	171.03		52.29
B	2,4-α-L-Rha*p*	H	5.17	4.04	3.84	3.6	3.76	1.14		
		C	98.43	76.11	68.65	75.11	67.28	16.92		
C	T-α-L-Rha*p*	H	4.63	3.84	3.66	3.33	3.93	1.14		
		C	100.61	71.27	69.22	71.75	69.21	16.92		
D	T-α-L-Ara*f*	H	5.13	4.1	3.91	4.02	3.72	3.62		
		C	109.18	82.01	76.69	83.86	60.96			
E	T-α-L-Ara*f*	H	5.04	4.02	3.84	3.94	3.72	3.62		
		C	107.09	80.99	76.95	83.84	60.96			
F	5-α-L-Ara*f*	H	4.99	4.03	3.84	4.11	3.7	3.78		
		C	107.43	80.87	76.69	81.82	66.2			
G	3,5-α-L-Ara*f*	H	5.01	4.19	3.99	3.93	3.84			
		C	107.32	79.12	82.22	83.97	66.04			
H	3,6-β-D-Gal*p*	H	4.43	3.54	3.64	4.01	3.81	3.82	3.92	
		C	103.14	69.67	80.05	69.93	73.33	69.26		
I	T-β-D-Gal*p*	H	4.38	3.25	3.44	3.18	3.6	--		
		C	103.21	72.77	74.93	81.7	74.36	62.38		
J	6-β-D-Gal*p*	H	4.37	3.44	3.55	3.82	3.94	3.92		
		C	103.25	70.6	72.66	73.59	68.84	69.24		
K	4-β-D-Gal*p*	H	4.54	3.57	3.66	4.06	3.6	3.67		
		C	104.33	72.09	73.33	77.77	74.93	60.89		
L	4-α-Gal*p*A	H	4.92	3.63	3.84	4.26	4.67			
		C	99.71	67.81	69.21	79.37	71.14	172.99		
M	4-β-Glc*p*A	H	4.56	3.43	3.65	3.47	3.80			
		C	104.00	74.46	75.73	79.02	76.35	171.70		

## Data Availability

Data sharing, not applicable. No new data were created or analyzed in this study. Data sharing is not applicable to this article.

## Supplementary Materials

The following supporting information can be downloaded at: https://www.mdpi.com/article/10.3390/nu15010080/s1, Figure S1. Sugar residues of DLP120; Figure S2. PMAAof DLP120; Figure S3. (**A**). The HPLC chromatogram of the crude polysaccharide of dandelion leaves (DLP120-1). (**B**) The HPLC chromatogram of the DLP120-1 after deproteinization and dialysis (DLP120-2). (**C**) The elution profile by Sephadex G-200 Purified; Figure S4. COSY (**a**), TOCSY (**b**) and the repeating unit (**c**) of DLP120; Table S1. Detailed parameters of NMR experiment of DLP120; Table S2. Summary of monosaccharide standard curve of DLP120 from ion chromatography information.

## References

[1] Siegel R.L., Miller K.D., Jemal A. (2019). Cancer statistics, 2019. CA Cancer J. Clin..

[2] Giovannucci E., Chan A.T. (2010). Role of Vitamin and Mineral Supplementation and Aspirin Use in Cancer Survivors. J. Clin. Oncol..

[3] Hou Y.-N., Deng G., Mao J.J. (2019). Practical Application of “About Herbs” Website Herbs and Dietary Supplement Use in Oncology Settings. Cancer J..

[4] Mehmood M.H., Malik A., Haider G., Akhtar M.S., Gilani A.H. (2017). Herbs for Health: An alternative approach to identify newer therapeutic option for the treatment of diabetes. Biochem. Pharmacol..

[5] Martinez M., Poirrier P., Chamy R., Prüfer D., Schulze-Gronover C., Jorquera L., Ruiz G. (2015). *Taraxacum officinale* and related species—An ethnopharmacological review and its potential as a commercial medicinal plant. J. Ethnopharmacol..

[6] Clare B.A., Conroy R.S., Spelman K. (2009). The Diuretic Effect in Human Subjects of an Extract of *Taraxacum officinale* Folium over a Single Day. J. Altern. Complement. Med..

[7] Schuetz K., Carle R., Schieber A. (2006). Taraxacum—A review on its phytochemical and pharmacological profile. J. Ethnopharmacol..

[8] El-Nagar D.M., Al-Dahmash B.A., Alkahtani S., Kalu A.A., Rady A. (2022). Dandelion (*Taraxacum officinale*) seeds extract attenuates hypercholesterolemia in swiss albino mice. J. King Saud Univ.-Sci..

[9] Hu C., Kitts D.D. (2005). Dandelion (*Taraxacum officinale*) flower extract suppresses both reactive oxygen species and nitric oxide and prevents lipid oxidation in vitro. Phytomedicine.

[10] Zhang Y., Hu Y.-F., Li W., Xu G.-Y., Wang K.-R., Li L., Luo H., Zou L., Wu J.-S. (2022). Updates and advances on pharmacological properties of Taraxacum mongolicum Hand.-Mazz and its potential applications. Food Chem..

[11] Hu C. (2018). Taraxacum: Phytochemistry and health benefits. Chin. Herb. Med..

[12] Davaatseren M., Hur H.J., Yang H.J., Hwang J.-T., Park J.H., Kim H.-J., Kim M.J., Kwon D.Y., Sung M.J. (2013). Taraxacum official (dandelion) leaf extract alleviates high-fat diet-induced nonalcoholic fatty liver. Food Chem. Toxicol..

[13] Mukherjee S., Jana S., Khawas S., Kicuntod J., Marschall M., Ray B., Ray S. (2022). Synthesis, molecular features and biological activities of modified plant polysaccharides. Carbohydr. Polym..

[14] Lis B., Rolnik A., Jedrejek D., Soluch A., Stochmal A., Olas B. (2019). Dandelion (*Taraxacum officinale* L.) root components exhibit anti-oxidative and antiplatelet action in an in vitro study. J. Funct. Foods.

[15] Wang L., Li T., Liu F., Liu D., Xu Y., Yang Y., Zhao Y., Wei H. (2019). Ultrasonic-assisted enzymatic extraction and characterization of polysaccharides from dandelion (*Taraxacum officinale*) leaves. Int. J. Biol. Macromol..

[16] Li F., Feng K.-L., Yang J.-C., He Y.-S., Guo H., Wang S.-P., Gan R.-Y., Wu D.-T. (2021). Polysaccharides from dandelion (Taraxacum mongolicum) leaves: Insights into innovative drying techniques on their structural characteristics and biological activities. Int. J. Biol. Macromol..

[17] Wu D.-T., He Y., Yuan Q., Wang S., Gan R.-Y., Hu Y.-C., Zou L. (2022). Effects of molecular weight and degree of branching on microbial fermentation characteristics of okra pectic-polysaccharide and its selective impact on gut microbial composition. Food Hydrocoll..

[18] Abuduwaili A., Rozi P., Mutailifu P., Gao Y., Nuerxiati R., Aisa H.A., Yili A. (2019). Effects of different extraction techniques on physicochemical properties and biological activities of polysaccharides from Fritillaria pallidiflora Schrenk. Process Biochem..

[19] Plaza M., Marina M.L. (2019). Pressurized hot water extraction of bioactives. TrAC Trends Anal. Chem..

[20] Getachew A.T., Cho Y.J., Chun B.S. (2018). Effect of pretreatments on isolation of bioactive polysaccharides from spent coffee grounds using subcritical water. Int. J. Biol. Macromol..

[21] Sakdasri W., Arnutpongchai P., Phonsavat S., Bumrungthaichaichan E., Sawangkeaw R. (2022). Pressurized hot water extraction of crude polysaccharides, β-glucan, and phenolic compounds from dried gray oyster mushroom. LWT.

[22] Lei J., Li W., Fu M.-X., Wang A.-Q., Wu D.-T., Guo H., Hu Y.-C., Gan R.-Y., Zou L., Liu Y. (2022). Pressurized hot water extraction, structural properties, biological effects, and in vitro microbial fermentation characteristics of sweet tea polysaccharide. Int. J. Biol. Macromol..

[23] Savag M.G., Lackman D.B., Smolens J. (1938). The isolation of the components of streptococcal nucleoproteins in serologically active form. J. Biol. Chem..

[24] Zhai X., Zhu C., Li Y., Zhang Y., Duan Z., Yang X. (2018). Optimization for pectinase-assisted extraction of polysaccharides from pomegranate peel with chemical composition and antioxidant activity. Int. J. Biol. Macromol..

[25] Blumenkrantz N., Asboe-Hansen G. (1973). New method for quantitative determination of uronic acids. Anal. Biochem..

[26] Bradford M.M. (1976). A rapid and sensitive method for the quantitation of microgram quantities of protein utilizing the principle of protein-dye binding. Anal. Biochem..

[27] Tian W.-T., Zhang X.-W., Liu H.-P., Wen Y.-H., Li H.-R., Gao J. (2020). Structural characterization of an acid polysaccharide from Pinellia ternata and its induction effect on apoptosis of Hep G2 cells. Int. J. Biol. Macromol..

[28] Miao Y.-Z., Lin Q., Cao Y., He G.-H., Qiao D.-R., Cao Y. (2011). Extraction of water-soluble polysaccharides (WSPS) from Chinese truffle and its application in frozen yogurt. Carbohydr. Polym..

[29] Su C.-H., Lai M.-N., Ng L.-T. (2017). Effects of different extraction temperatures on the physicochemical properties of bioactive polysaccharides from Grifola frondosa. Food Chem..

[30] Wang L.B., Gao J.Y., Li L.Y., Huang J., Yang Y., Xu Y.Q., Wang Y.B., Liu Y. (2021). Characterization and Biological Activities of Polysaccharides from Dandelion (*Taraxacum offificinale*) Leaves. Starch-Starke.

[31] Zhang S.J., Song Z.T., Shi L.J., Zhou L.A., Zhang J., Cui J.L., Li Y.H., Jin D.Q., Ohizumi Y., Xu J. (2021). A dandelion polysaccharide and its selenium nanoparticles: Structure features and evaluation of anti-tumor activity in zebrafish models. Carbohydr. Polym..

[32] Chen Y., Xie M.-Y., Nie S.-P., Li C., Wang Y.-X. (2008). Purification, composition analysis and antioxidant activity of a polysaccharide from the fruiting bodies of Ganoderma atrum. Food Chem..

[33] Gong G., Zhao J., Wang C., Wei M., Dang T., Deng Y., Sun J., Song S., Huang L., Wang Z. (2018). Structural characterization and antioxidant activities of the degradation products from Porphyra haitanensis polysaccharides. Process Biochem..

[34] Yang L., Zhang L.-M. (2009). Chemical structural and chain conformational characterization of some bioactive polysaccharides isolated from natural sources. Carbohydr. Polym..

[35] Sims I.M., Carnachan S.M., Bell T.J., Hinkley S.F.R. (2018). Methylation analysis of polysaccharides: Technical advice. Carbohydr. Polym..

[36] Kang J., Cui S.W., Phillips G.O., Chen J., Guo Q., Wang Q. (2011). New studies on gum ghatti (Anogeissus latifolia) Part III: Structure characterization of a globular polysaccharide fraction by 1D, 2D NMR spectroscopy and methylation analysis. Food Hydrocoll..

[37] Kim J.S., Reuhs B.L., Michon F., Kaiser R.E., Arumugham R.G. (2006). Addition of glycerol for improved methylation linkage analysis of polysaccharides. Carbohydr. Res..

[38] Guo Q.B., Cui S.W., Kang J., Ding H.H., Wang Q., Wang C. (2015). Non-starch polysaccharides from American ginseng: Physicochemical investigation and structural characterization. Food Hydrocoll..

[39] Wang N.F., Zhang X.J., Wang S.W., Guo Q.B., Li Z.J., Liu H.H., Wang C.L. (2020). Structural characterisation and immunomodulatory activity of polysaccharides from white asparagus skin. Carbohydr. Polym..

[40] do Prado S.B.R., Santos G.R.C., Mourão P.A.S., Fabi J.P. (2019). Chelate-soluble pectin fraction from papaya pulp interacts with galectin-3 and inhibits colon cancer cell proliferation. Int. J. Biol. Macromol..

[41] Makarova E.N., Shakhmatov E.G. (2021). Characterization of pectin-xylan-glucan-arabinogalactan proteins complex from Siberian fir Abies sibirica Ledeb. Carbohydr. Polym..

[42] Wu D.-T., He Y., Fu M.-X., Gan R.-Y., Hu Y.-C., Peng L.-X., Zhao G., Zou L. (2022). Structural characteristics and biological activities of a pectic-polysaccharide from okra affected by ultrasound assisted metal-free Fenton reaction. Food Hydrocoll..

[43] Huang L., Zhao J., Wei Y., Yu G., Li F., Li Q. (2021). Structural characterization and mechanisms of macrophage immunomodulatory activity of a pectic polysaccharide from Cucurbita moschata Duch. Carbohydr. Polym..

[44] Makarova E.N., Shakhmatov E.G. (2020). Structural characteristics of oxalate-soluble polysaccharides from Norway spruce (Picea abies) foliage. Carbohydr. Polym..

[45] Yue F., Xu J., Zhang S., Hu X., Wang X., Lü X. (2022). Structural features and anticancer mechanisms of pectic polysaccharides: A review. Int. J. Biol. Macromol..

[46] Caffall K.H., Mohnen D. (2009). The structure, function, and biosynthesis of plant cell wall pectic polysaccharides. Carbohydr. Res..

[47] Feng Y.-Y., Ji H.-Y., Dong X.-D., Liu A.-J. (2019). An alcohol-soluble polysaccharide from Atractylodes macrocephala Koidz induces apoptosis of Eca-109 cells. Carbohydr. Polym..

[48] de Camargo M.R., Frazon T.F., Inacio K.K., Smiderle F.R., Amôr N.G., Dionísio T.J., Santos C.F., Rodini C.O., Lara V.S. (2022). Ganoderma lucidum polysaccharides inhibit in vitro tumorigenesis, cancer stem cell properties and epithelial-mesenchymal transition in oral squamous cell carcinoma. J. Ethnopharmacol..

[49] Zhong S., Ji D.-F., Li Y.-G., Lin T.-B., Lv Z.-Q., Chen H.-P. (2013). Activation of P27kip1-cyclin D1/E-CDK2 pathway by polysaccharide from Phellinus linteus leads to S-phase arrest in HT-29 cells. Chem.-Biol. Interact..

[50] El-Emam S.Z., Abo El-Ella D.M., Fayez S.M., Asker M., Nazeam J.A. (2021). Novel dandelion mannan-lipid nanoparticle: Exploring the molecular mechanism underlying the potent anticancer effect against non-small lung carcinoma. J. Funct. Foods.

[51] Khotimchenko M. (2020). Pectin polymers for colon-targeted antitumor drug delivery. Int. J. Biol. Macromol..

[52] Dick-Perez M., Wang T., Salazar A., Zabotina O.A., Hong M. (2012). Multidimensional solid-state NMR studies of the structure and dynamics of pectic polysaccharides in uniformly 13C-labeled Arabidopsis primary cell walls. Magn. Reson. Chem..

[53] Maxwell E.G., Colquhoun I.J., Chau H.K., Hotchkiss A.T., Waldron K.W., Morris V.J., Belshaw N.J. (2015). Rhamnogalacturonan I containing homogalacturonan inhibits colon cancer cell proliferation by decreasing ICAM1 expression. Carbohydr. Polym..

[54] Zhang S., He F., Chen X., Ding K. (2019). Isolation and structural characterization of a pectin from Lycium ruthenicum Murr and its anti-pancreatic ductal adenocarcinoma cell activity. Carbohydr. Polym..

[55] Wang S., Li P., Lu S.-M., Ling Z.-Q. (2016). Chemoprevention of Low-Molecular-Weight Citrus Pectin (LCP) in Gastrointestinal Cancer Cells. Int. J. Biol. Sci..

[56] Azzopardi M., Farrugia G., Balzan R. (2017). Cell-cycle involvement in autophagy and apoptosis in yeast. Mech. Ageing Dev..

[57] Prado S.B.R.d., Ferreira G.F., Harazono Y., Shiga T.M., Raz A., Carpita N.C., Fabi J.P. (2017). Ripening-induced chemical modifications of papaya pectin inhibit cancer cell proliferation. Sci. Rep..

[58] Cheng H., Zhang Z., Leng J., Liu D., Hao M., Gao X., Tai G., Zhou Y. (2013). The inhibitory effects and mechanisms of rhamnogalacturonan I pectin from potato on HT-29 colon cancer cell proliferation and cell cycle progression. Int. J. Food Sci. Nutr..

[59] Zhang T., Miller M.C., Zheng Y., Zhang Z., Xue H., Zhao D., Su J., Mayo K.H., Zhou Y., Tai G. (2017). Macromolecular assemblies of complex polysaccharides with galectin-3 and their synergistic effects on function. Biochem. J..

[60] Fortuna-Costa A., Gomes A.M., Kozlowski E.O., Stelling M.P., Pavão M.S.G. (2014). Extracellular Galectin-3 in Tumor Progression and Metastasis. Front. Oncol..

[61] Pfeifer L., Baumann A., Petersen L.M., Höger B., Beitz E., Classen B. (2021). Degraded Arabinogalactans and Their Binding Properties to Cancer-Associated Human Galectins. Int. J. Mol. Sci..

[62] Zhang T., Zheng Y., Zhao D., Yan J., Sun C., Zhou Y., Tai G. (2016). Multiple approaches to assess pectin binding to galectin-3. Int. J. Biol. Macromol..

[63] Cui L., Wang J., Huang R., Tan Y., Zhang F., Zhou Y., Sun L. (2019). Analysis of pectin from Panax ginseng flower buds and their binding activities to galectin-3. Int. J. Biol. Macromol..

