# Supplementary material for: Structural Characteristic and In-Vitro Anticancer Activities of Dandelion Leaf Polysaccharides from Pressurized Hot Water Extraction
Source: Nutrients. 2022 Dec 24;15(1):80. doi: 10.3390/nu15010080 (PMC9824204; doi:10.3390/nu15010080)
Supplement: Supplementary file 1 [file nutrients-15-00080-s001.zip › nutrients-2056079-supplementary.pdf]

Supplementary Materials

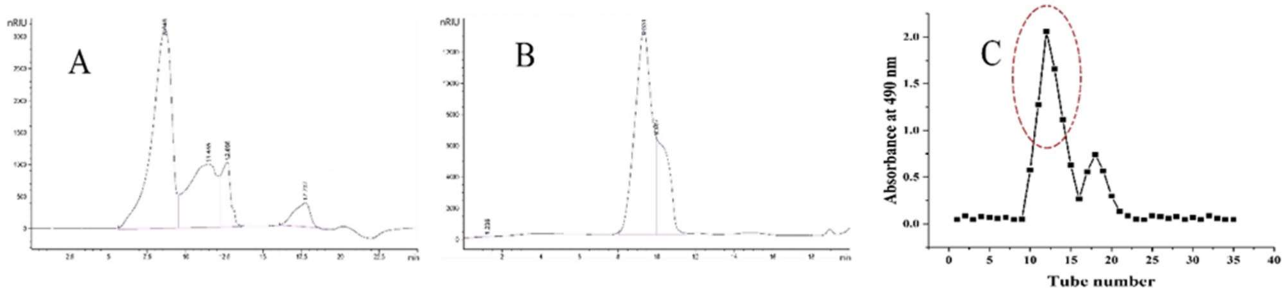

Figure S1. (**A**). The HPLC chromatogram of the crude polysaccharide of dandelion leaves (DLP120-1). (**B**) The HPLC chromatogram of the DLP120-1 after deproteinization and dialysis (DLP120-2). (**C**) The elution profile by Sephadex G-200 Purified.

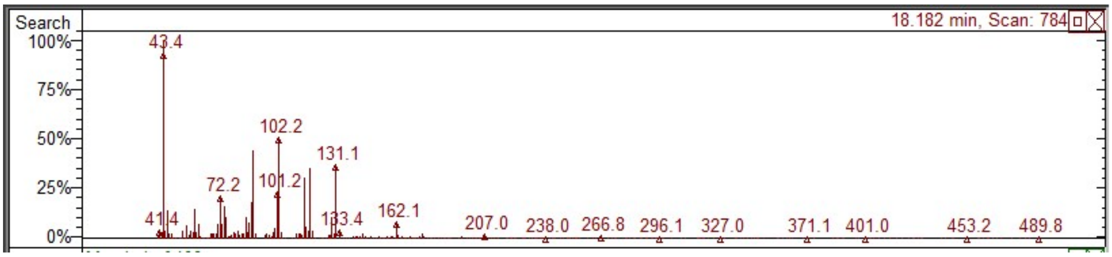

A

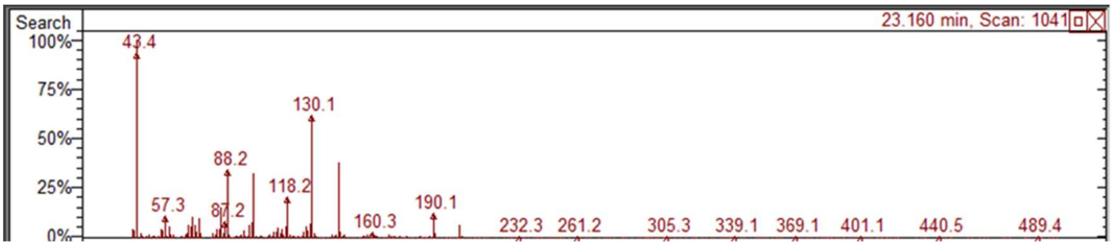

B

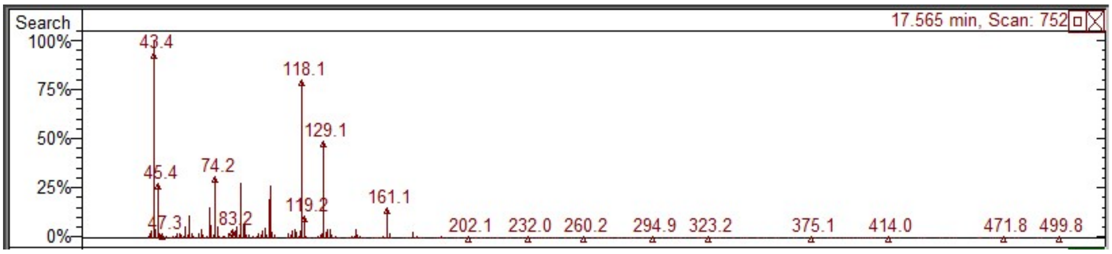

C

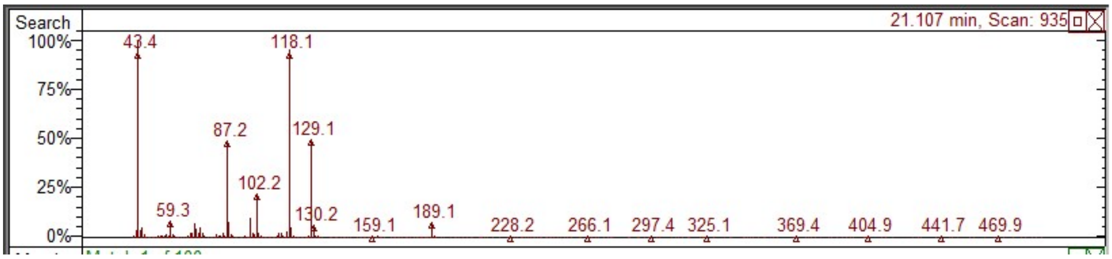

D

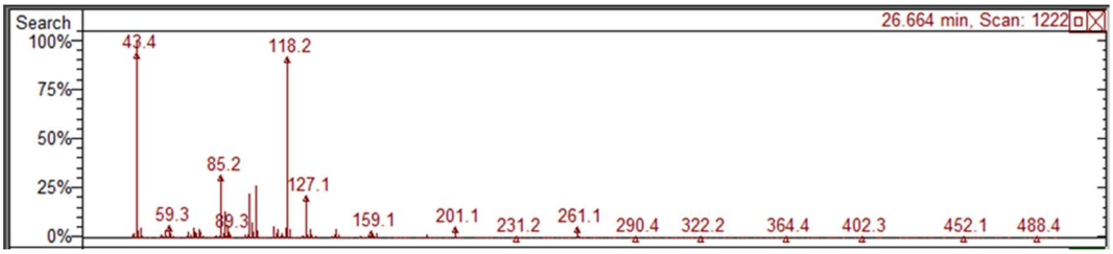
Search
26.664 min, Scan: 1222
100%
75%
50%
25%
0%
43.4
118.2
85.2
127.1
59.3
89.3
159.1
201.1
231.2
261.1
290.4
322.2
364.4
402.3
452.1
488.4

E

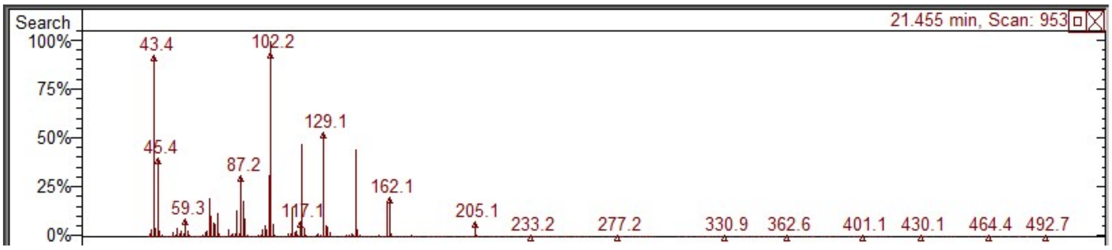
Search
21.455 min, Scan: 953
100%
75%
50%
25%
0%
43.4
102.2
129.1
45.4
87.2
162.1
59.3
117.1
205.1
233.2
277.2
330.9
362.6
401.1
430.1
464.4
492.7

F

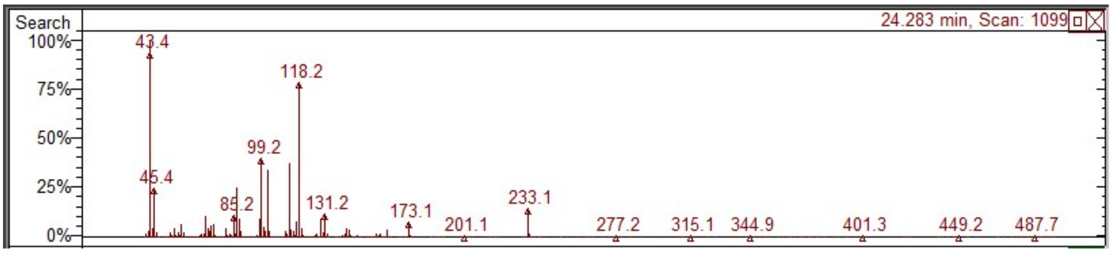
Search
24.283 min, Scan: 1099
100%
75%
50%
25%
0%
43.4
118.2
99.2
45.4
85.2
131.2
173.1
233.1
201.1
277.2
315.1
344.9
401.3
449.2
487.7

G

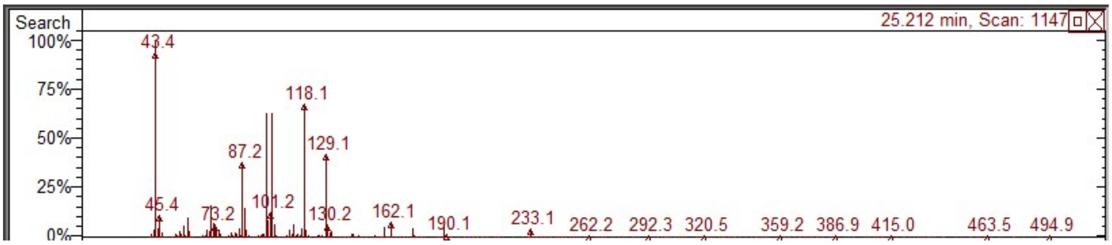
Search
25.212 min, Scan: 1147
100%
75%
50%
25%
0%
43.4
118.1
129.1
87.2
45.4
73.2
101.2
130.2
162.1
190.1
233.1
262.2
292.3
320.5
359.2
386.9
415.0
463.5
494.9

H

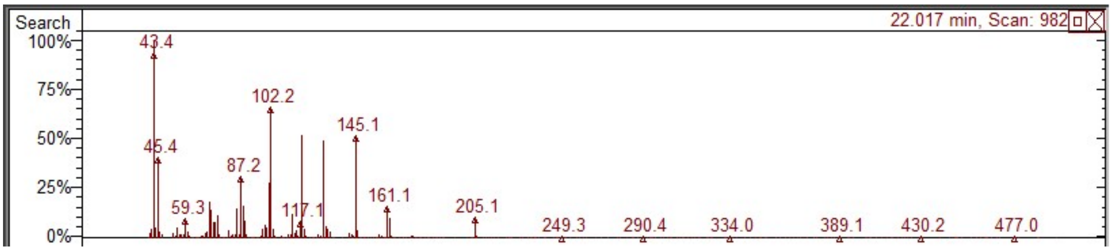
Search
22.017 min, Scan: 982
100%
75%
50%
25%
0%
43.4
102.2
145.1
45.4
87.2
59.3
117.1
161.1
205.1
249.3
290.4
334.0
389.1
430.2
477.0

I

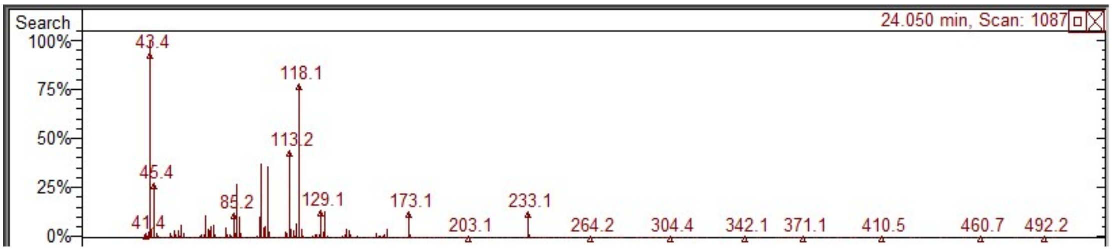
Search
24.050 min, Scan: 1087
100%
75%
50%
25%
0%
43.4
118.1
113.2
45.4
41.4
85.2
129.1
173.1
233.1
203.1
264.2
304.4
342.1
371.1
410.5
460.7
492.2

J

Figure S2. Sugar residues of DLP120. (A). T-Rha*p* (B). 2,4-Rha*p* (C). T-Ara*f* (D). 5-Ara*f* (E). 3,5-Ara*f* (F). T-Gal*p* (G). 4-Gal*p*/Gal*p*A (H). 6-Gal*p* (I). T-Glc*p* (J). 4-Man*p*

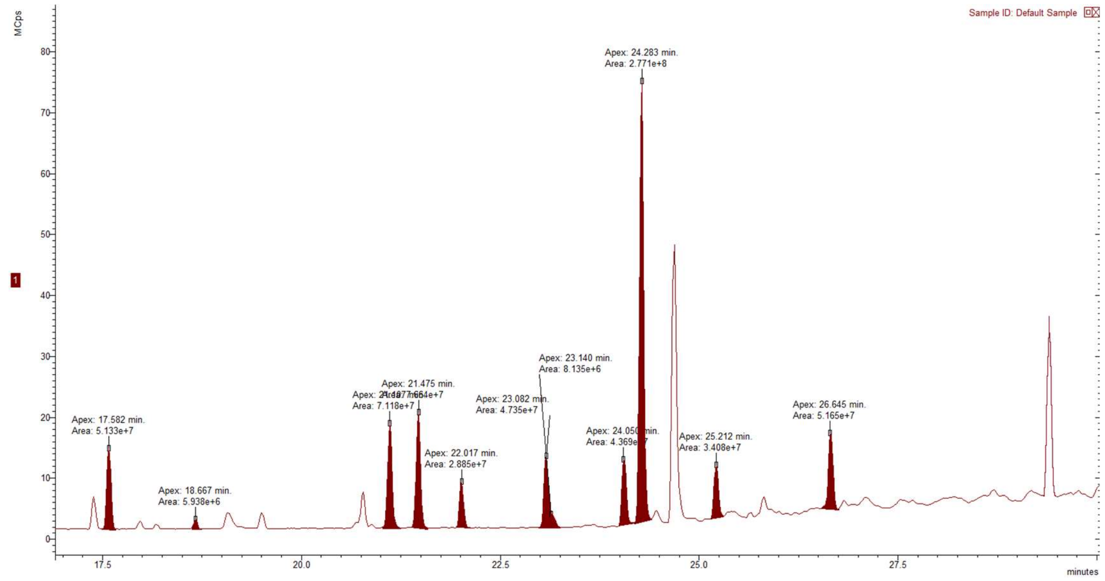

PMAA

Figure S3. PMAAof DLP120.

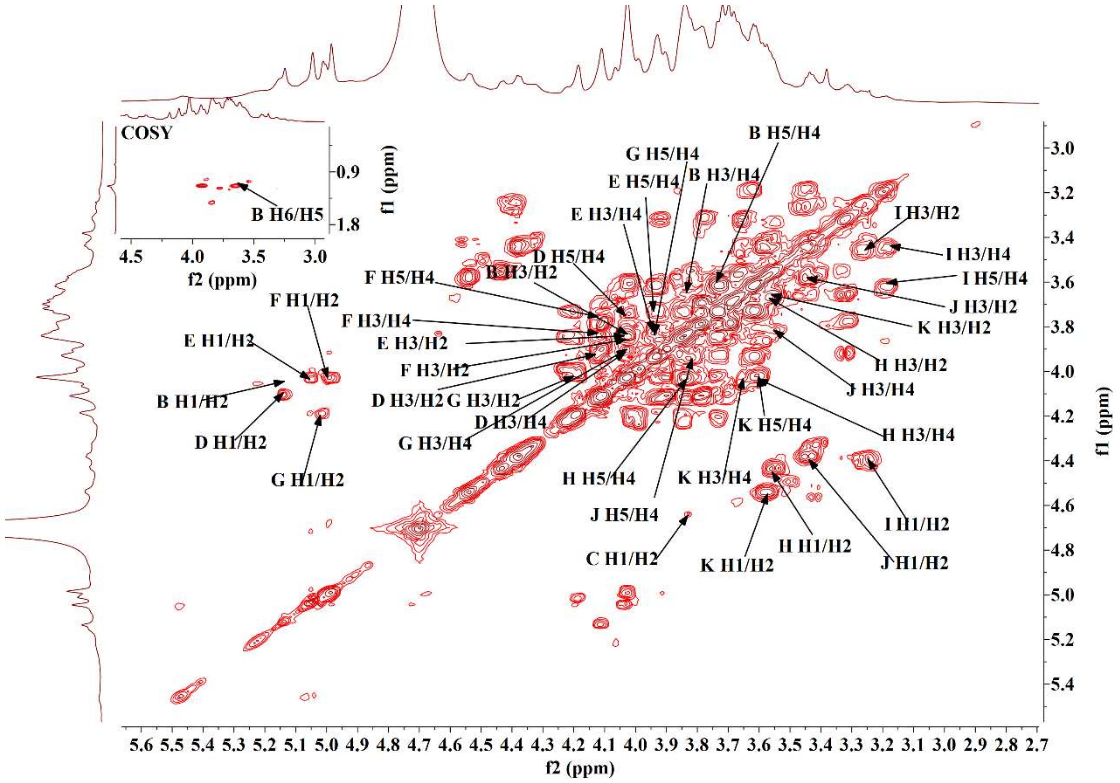

a

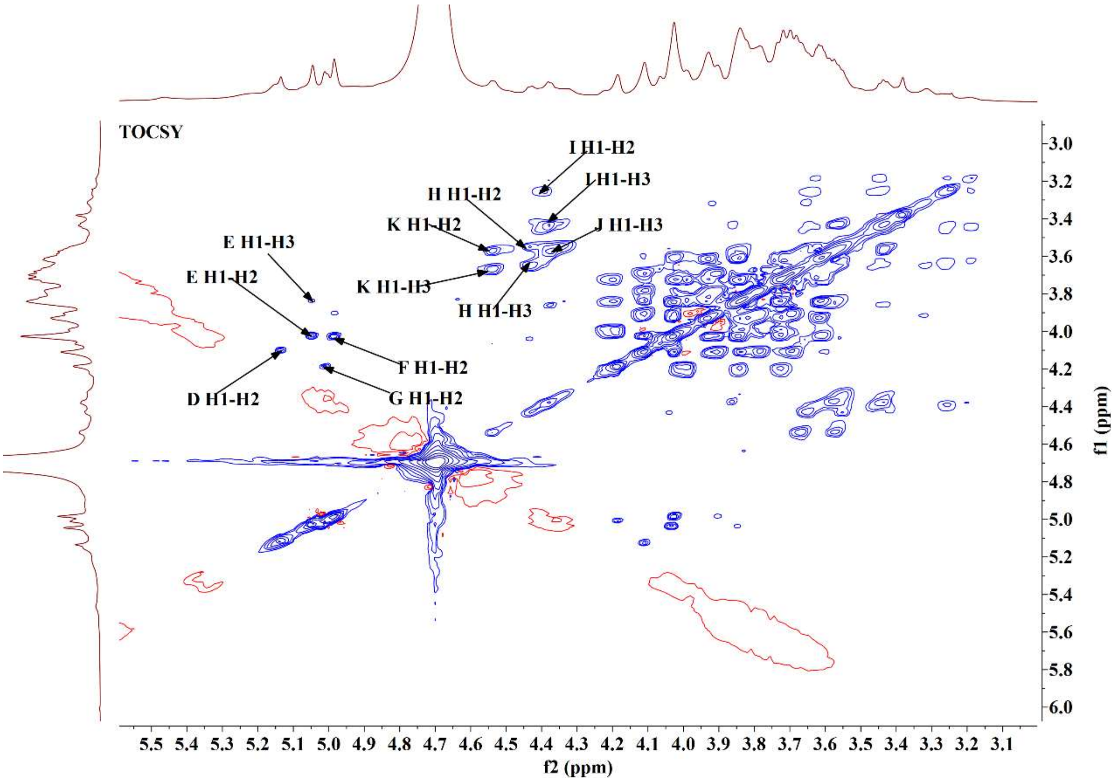

b

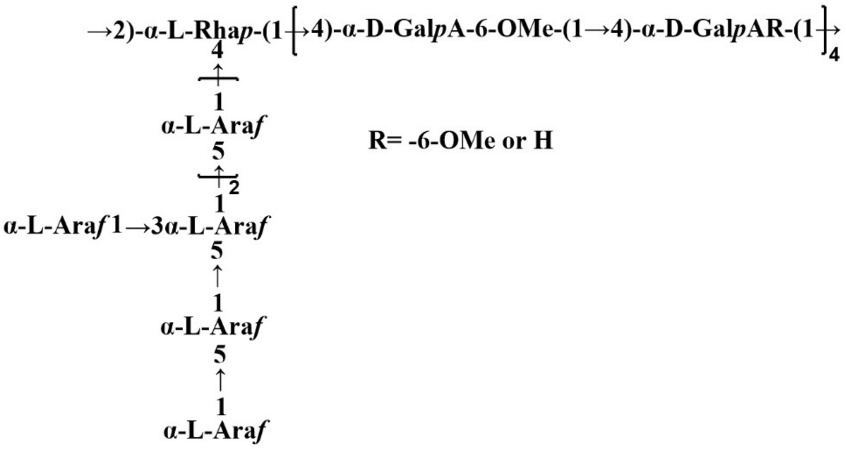

c

Figure S4. COSY (**a**), TOCSY (**b**) and the repeating unit (**c**) of DLP120.

Table S1. Detailed parameters of NMR experiment of DLP120.

Type of NMR	Pulprog	Acquisition time (AQT)	Time domain (TD)	Number of scans(NS)	Delays(D1)	Temperature(T)
^{13}C	zgpg30	0.7689557 s	65536	7600	2 s	298 k
^{1}H	zg30	2.3243434 s	65536	64	1 s	298 k
COSY	cosygpmfqf	0.1331200 s	2048	32	0.08 s	298 k
TOCSY	mlevphpp	0.1331200 s	2048	48	2 s	298 k
HSQC	hsqcetgpssp2.2	0.1331200 s	2048	46	2 s	298 k
HMBC	hmbcgpndqf	0.1331200 s	2048	58	5 s	298 k

Table S2. Summary of monosaccharide standard curve of DLP120 from ion chromatography information.

Sample Name	Peak time (min)	Slope	Fitting degree
Rha	8.53	0.3043	0.99136
Ara	10.28	0.5869	0.99427
Gal	13.31	0.7344	0.99703
Glc	14.27	0.8649	0.99632
Man	15.94	0.5022	0.99527
GalA	25.70	0.1643	0.99860
GlcA	26.44	0.2831	0.99744
